# Psychotic Arousal and the Psychopathology of Acute Schizophrenia: An Exploratory Study of the Experiential Emotional State in Acute Psychosis

**DOI:** 10.3390/jcm13185477

**Published:** 2024-09-15

**Authors:** Maria M. Margariti, Ilias I. Vlachos, Dimitra Mpourazana, Panagiotis Aristotelidis, Mirjana Selakovic, Maria Ifanti, Charalambos Papageorgiou

**Affiliations:** 1 1st Department of Psychiatry, Medical School, National and Kapodistrian University of Athens, 11528 Athens, Greece; 2 2nd Department of Psychiatry, Medical School, National and Kapodistrian University of Athens, 11527 Athens, Greece; 3 Department of Psychiatry, “Sismanogleio” General Hospital, 15126 Athens, Greece; 4 University Mental Health Research Institute, 11527 Athens, Greece

**Keywords:** psychotic arousal scale, emotional arousal, psychotic experiences, abnormal subjective experiences, abnormal experiential feelings, psychotic emergence

## Abstract

**Background**: Increasing research data suggest that the dysfunction of emotional brain systems may be an important contributor to the pathophysiology of schizophrenia. However, contemporary psychopathology consistently underestimates the role of emotions in the phenomenology of the disease. Psychotic arousal (PA) is a conceptually defined psychopathological construct aiming to portray the experiential emotional state of acute psychosis. The concept provides an explanatory model for the emergence of psychosis, and the formation and maintenance of delusions based on neurobiological models on the formation of core consciousness and subjectivity. This is the first exploratory study of the major assumptions, endorsed in the project summarized as follows: (1) psychotic arousal is a discrete state, eligible for investigation; (2) abnormal experiential feelings are an integral part of this state; and (3) the state is responsive to antipsychotic intervention during the first weeks of treatment. **Methods**: We developed the Psychotic Arousal Scale (PAS) accordingly, explored its first psychometric properties and tested its relation to other psychopathological measures. Fifty-five acute schizophrenia patients were evaluated with the PAS, the Positive and Negative Syndrome Scale, the Brown Assessment of Beliefs Scale, the Hamilton Anxiety Scale, and the Calgary Depression Scale. Cronbach α coefficients, *t*-test analysis, correlations and mixed linear regression models were applied for testing the internal reliability of the scale, associations between parameters and sensitivity to change in three time periods during therapeutic intervention. **Results**: The results of the study support that (PA) is eligible for investigation as a discrete psychopathological state. Abnormal experiential feelings are an integral part of this state, presenting high affinity with other affective measures; their degree of severity relates to the delusions’ conviction and are amenable to antipsychotics early in treatment during the acute psychotic episode. **Conclusions**: The findings of this exploratory study are connotative of the presence of an emotional arousal permeated by abnormal experiential feelings during acute psychosis, largely overlooked by contemporary psychopathology.

## 1. Introduction

Schizophrenia is a mental disorder, often resulting in severe functional disability and cognitive impairment, including intellectual abilities such as perception and reasoning [1]. Prevalent symptoms are mainly characterized by disorganization, as well as thought and perceptual disturbances, such as delusions and hallucinations, which are known as positive symptoms of schizophrenia [2,3]. There is also a widespread conceptualization of schizophrenia as a primarily neurocognitive disorder, involving mainly a dysfunction of the neural and cognitive systems subserving reasoning, memory, language and perception [4,5].

However, concerns have been raised regarding the lack of specific neurocognitive impairments characterized in patients with schizophrenia [6,7,8]. The presence of considerable variability in cognitive function and heterogeneity in the magnitude of the impairment across subjects suggests a non-uniform cognitive deficit [9,10].

In addition, there is evidence identifying important nonspecific mediators of neurocognitive test performance, some of which can be accounted for in trials, including motivation, effort, defeatist performance beliefs, stress, anxiety and physical inactivity [11]. 

Though neurocognitive dysfunction is a prevalent characteristic of disease, it remains unclear so far, how these cognitive impairments constitute an explanatory framework for the core and defining symptoms of psychosis. Increasing research data in recent years suggests that the dysfunction of emotional brain systems may be at least equally important in understanding the disorder and providing the tools for a unified explanatory system of its pathophysiology.

### 1.1. Emotions and Schizophrenia

#### 1.1.1. Negative Symptoms

Emotion and cognition can best be thought of as separate but interacting mental functions, mediated by separate but interacting brain systems [12,13,14,15].

Since the initial descriptions of schizophrenia, emotional symptoms such as lack of emotional responsivity, loss of interest and inability to “feel” for others were observed in the patients, and were recognized as central features of the disorder [16]. Nevertheless, their identification and quantification proved to be, in practice, a challenging task and diverted psychiatric attention—as expressed by Schneiderian first-rank symptoms—to more pronounced, peculiar and easily identifiable symptoms [17]. Today, blunted affect, alogia, asociality, anhedonia and avolition correspond to the so-called negative symptoms, labeled as such because they involve deficits in something that is typically present among healthy people [18].

According to several studies, negative symptoms can be explained by two factors: diminished expression (blunted affect), and diminished motivation and pleasure (avolition, anhedonia, asociality) [19,20,21]. Both factors apparently involve deficits in emotion and, specifically, diminished expression pertains to a deficit in the outward expression of emotion via the face and voice, while diminished motivation and pleasure are related to a deficit in pleasure and goal-directed behavior across social, work and recreational life domains [20,22]. Nevertheless, there is consistent evidence that patients with schizophrenia report experiencing similar amounts of emotion to people without schizophrenia [23], and convergent findings indicate the ability of people with schizophrenia to provide reliable and valid reports of their emotional experience, even when assessments occur across changes in symptoms and medication status [24,25].

Negative symptoms may be present early in the course of illness, before an acute psychotic episode, leading to the clinical diagnosis of schizophrenia; they persist over time, increase in severity and remain between acute episodes of illness, therefore presenting a “trait” characteristic in the course of illness. According to some findings, their early appearance predicts the eventual first psychotic episode [26,27,28]. Considering all of the above, Kring [29] suggests that negative symptoms possibly have a causal importance in schizophrenia.

Several studies have also documented the independence of negative from positive symptoms, as well as their non- responsiveness to antipsychotics, further suggesting independent underlying pathophysiological processes [26,30].

#### 1.1.2. Emotions and the Emergence of Schizophrenia

##### The Pre-Delusional Emotional State

There is also another emotional state relevant to the emergence of acute psychotic state in schizophrenia, largely neglected by contemporary descriptive psychopathology.

Early investigators have specifically described a phenomenon named “delusional mood”, and ascribed it to the earlier phases of illness, linking it to the genesis of delusions. Delusional mood is referring to a diffuse affective state preceding the formation of primary delusions, permeated by a variety of derealization and depersonalization feelings, as well as subtle experiences of the self and world transformation, which eventually may—or may not—evolve to a full-blown psychotic episode. Affective symptoms of this state include an increasing affective tone and tension, free-floating anxiety, guilt and depression, but also elation or ecstasy. According to descriptions, the delusional mood, as it evolves, becomes increasingly self-referential; “whatever is going on or about to happen is directly linked to the patient”. Experiences of self-reference are regularly an inherent part of the clinical picture of delusional mood, and “primary” in the sense that there is an immediately a sensed link between the patient and others that is psychologically irreducible [31].

Matussek [32] considered that idiosyncratic, self-referential meanings are derived from the emergence of normally unnoticeable properties of objects in the perceptual field, and that delusional meaning is already inherent in perception itself.

Fuchs [33], referring to his own patients, mentions the following: “A schizophrenic patient of mine, watching the cars on the street, suddenly felt ‘something metallic’ of the coachwork leap at him and merge with him. He felt ‘kind of hard, sharp and cold’, like the car itself and its contours. Another patient felt an ‘energetic potential’ passing over from other persons to his body and entering through his forehead, especially when he was being looked at. He then had to walk for some time to let this tension flow off to the ground again”.

In the delusional mood (Wahnstimmung), according to Jaspers [34], patients “feel uncanny [‘unheimlich’] … everything gets a new meaning. The environment is somehow different”. Jaspers specifies, though, that perceptual content remains unchanged in itself.

Bleuler [35] emphasized the “affective” nature of delusional mood and proposed that an abnormal affect disrupts the processing of information and linearity of logical thinking, thereby facilitating the formation of delusions.

Hagen [36] also notes that “the disturbed affect may not be recognized, not only because patients are able to control themselves or because doctors do not always investigate this possibility closely enough, but also because the severity of the delusion itself may tend to mask or distract from the disturbed affect.”

Delusional mood is imbued by feelings of experiential alienation and altered modalities of world-oriented and self-oriented experience that precede and ground delusions [37]. Jaspers [34], regarding abnormal experiences, mentions the following: “If we try to get some closer understanding of these primary experiences of delusion, we soon find we cannot really appreciate these quite alien modes of experience. They remain largely incomprehensible, unreal and beyond our understanding. Yet some attempts have been made. We find that there arise in the patient certain primary sensations, vital feelings, moods, awareness”.

However, Kurt Schneider [17] stated that “delusional perception”, referring to a sudden, self-referential delusion triggered by neutral perceptual content, is a disorder of thought and an abnormal interpretation of an intact perception. For Schneider, a delusional atmosphere, though often preceding, is very vague and can offer no content pointing to the delusional perception that ensues later [38].

Regardless of how delusional mood and its altered experiential condition guide to the formation of “true” delusions in schizophrenia, the phenomenon is traditionally recognized as an exclusively pre-delusional state [38]. “When delusions are present it no longer makes sense to speak of delusional mood” [31].

This unverified consensus—as we will try to explain below—limits accessibility to an explanatory framework for the emergence of acute psychosis. Several important questions urge for answers, such as the following: (a) What are the nature and the role of this complex state in the emergence of psychosis; (b) How exactly does it switch to the evolution of full-blown psychosis; (c) What is the nature of these “experiences” and how do they connect to the underlying affective state; and (d) How do primary delusions—seen exclusively as disorders of thought—relate to this state with such a decisive, undoubtful and self-evidential way?

##### The Emotional State of Acute Schizophrenia: Clinical Observations

In contemporary descriptive psychopathology, the emotional state of the patient at the time florid psychosis emerges is overshadowed and treated as a confounding noise in the face of more interesting morbid manifestations such as delusions and/or hallucinations.

However, an acute psychotic state is dominated by an affective and emotional component that appears much more discrete, though rather elusive, than that observed in merely affective and anxiety disorders [39,40,41,42]. Affective symptoms, coloring typically the acute state, are easily identifiable in behavior and emotional expressions, yet are subjectively experienced and overwhelming for the patients. Symptoms may include a vast array, comprising not only anxiety, excitement or tension, but also fear, anguish, perplexity, guilt, sorrow, puzzlement and ecstasy [43], even though these conventional affective classifications appear opaque and diffuse, and can hardly be defined with precision by the patients themselves. In contemporary descriptive psychopathology, these symptoms are traditionally regarded as peripheral or incidental during an acute psychotic episode [44], and are implicitly attributed to a secondary affective response to positive symptoms, as opposed to principal affective symptoms constituting the negative syndrome in schizophrenia.

However, the clinical investigation of this emotional state and affective symptoms during the psychopharmacological treatment of an acute psychotic episode indicates that these affective symptoms respond more rapidly to psychopharmacological treatment than core psychotic symptoms, suggesting that they are not secondary in nature [45,46,47,48,49,50]. Indeed, the action of antidopaminergic agents has been attributed to dampening the emotional symptoms and distress to which symptoms occupy the mind, and “it is only later, over the ensuing weeks, that the fundamental content of the delusions and hallucinations is deconstructed and (only for some) recedes entirely from awareness” [51,52,53]. Furthermore, thorough clinical scrutiny reveals that the diffuse affective state pertained in the acute state of schizophrenia is accompanied by similar feelings of experiential alienation and abnormal experiences as they appear in the pre-delusional state, though in varying intensities and frequencies. It is difficult to distinguish these abnormal experiences from their ideational envelope at the peak of the acute state, but they are easily accessible at first, as soon as the emotional turbulence decreases in intensity and as soon as delusions start to fade out, to allow for the cooperation and self-reflection of the patient. Questions such as “What has changed and why don’t you bother any more so much with your prior concerns” or “Why have you started to question their validity now”, typically elicit answers such as “because I don’t feel it anymore”, and lead patients to promptly complement descriptions of their experienced abnormal feelings at the time of the emotional commotion [54]. They describe in detail feelings of sudden self-relevance to trivial events or insignificant surrounding objects, feelings of alienation and transformation of the self and the world, and losing the boundaries of the self or of the ownership of thoughts, movements or parts of the body, intertwined and often combined to each other. These feelings appear in an instantaneous and random way ‘every here and now’ the person interacts with internal or external events or objects. These feelings barrage and overwhelm patients during acute psychosis in increasing frequency and intensity. However, not all these experiences seem able to distort thought toward delusion, as it seems that some conditions must be required. 

Under the influence of experiences, delusions expand and evolve. Indeed, delusions in the acute state of schizophrenia are not formed as a static and integral scenario but develop and spread in a wavy manner. It is only at the time of amelioration of tension/uneasiness and “felt” experiences that delusions do not expand or florid further. Furthermore, it is only after the eventual cessation of “felt experiences” in response to antipsychotic treatment that the patient can reconsider the delusions. Meticulous investigation of the degree of awareness of the symptoms indicates that patients at the time of amelioration of the affective turmoil usually declare that critical symptoms (e.g., the delusional content of surveillance/passivity/control or referential content) are no longer valid, but very often, they insist without doubt that several days prior—when the affective turmoil was present—they were! A phenomenon well described by Amador [55] in his approach to ‘insight into illness as a multidimensional construct’.

#### 1.1.3. Toward an Emotional Continuum in the Emergence of Psychosis

Based on the above observations, we argue that the emotional state, described as strictly a “predelusional state” with its adjacent abnormal experiences, does not disappear in some mysterious way as delusions appear in the clinical picture. Delusions may offer a redeeming answer to abnormal experiences—as claimed before [56]— but the acute phase of schizophrenia resembles more a boiling cauldron or a volcano ready to explode than a relative calming, rationalization phase following a stormy period. Contrary to implicit assumptions, it seems that this complex emotional state, with its abnormal experiences, does not only not disappear as the acute phase of psychosis emerges, but it is its unremitting and increasingly intensified presence, as long as the acute phase persists, that shapes the absurd core content of the delusions and expands them further. Fayaerts et al. [57] also stressed the primacy of these experiences in the formation of the delusions and their “endured presence throughout the psychotic episode and even after its remission”.

#### 1.1.4. Neurobiological Data on Emotions in Florid Psychosis

Although contemporary psychopathology underestimated the role of emotions and the affects during the emergence and acute psychotic state, neurobiological studies in recent years provide accumulated evidence regarding the contribution of emotional systems in the development of acute schizophrenia. The mesolimbic dopamine system has been seen as a critical component in the “attribution of salience”, a process whereby events and thoughts come to grab attention, drive action and influence goal-directed behavior. Schizophrenia patients presenting with positive symptoms evidence high levels of emotional arousal, a dysfunction with “state” characteristics, that has been linked to corticolimbic hyperactivation and an impaired processing of emotional salience (i.e., increased emotional response to non-emotional stimuli) [58,59,60].

Evidence obtained from in vivo brain imaging, post-mortem and rodent studies suggests that the medial temporal lobe and its individual structures are extensively implicated in schizophrenia pathophysiology [61], and that amygdalocortical circuitry, the anterior cingulate cortex and hippocampus—key components of the limbic lobe—are an important focus for the study of schizophrenia, and may reflect clinical dysfunction overall [62].

F-MRI studies also indicate that the disruption of emotional brain systems may represent an important biological substrate for the pathophysiology of early psychosis and ultra-high-risk states. Modinos et al. [63] specifically reported an association between neural abnormalities during aberrant emotional salience, subjective experience and psychotic symptoms, demonstrating a link with positive symptoms of the illness in first-episode-psychosis patients.

Pinkham at al. [64] presented findings identifying increased resting cerebral blood flow in the amygdala in paranoid compared with non-paranoid individuals with schizophrenia, and suggest that amygdala hyperactivity at rest may subserve paranoid ideation. Resent findings [65] also implicate the limbic circuit pathology to active delusions of persecution in schizophrenia patients with increased connectivity between hippocampus and amygdala compared to patients without paranoia. Paranoia severity was also linked to increased connectivity between the hippocampus and amygdala. Meng Zhang at al. [66] found that patients with first-episode schizophrenia had abnormal functional connectivity in the amygdala subregions, and the altered resting-state functional connectivity was associated with positive symptoms. The authors concluded the presence of disruptive resting-state functional connectivity patterns of the amygdala subregional–sensorimotor networks in first-episode schizophrenia.

Aleman [12] emphasizes the presence of the so called “emotional paradox” in schizophrenia characterized by a reduction in emotional perception and expression of the face of increased subjective emotional arousal and reactivity. He underlines the disjunction between the “negative emotional symptoms” (reduced emotional expression and ability to recognize emotions) and “positive emotional symptoms” (excess of emotion, subjective emotional arousal and restlessness) concomitant to an acute psychotic state. He further proposes a “two-hit” model of amygdala abnormalities implying two key abnormalities: (1) a structural lesion of the amygdala, and (2) elevated levels of dopamine in the amygdala to explain the “emotional paradox”.

### 1.2. Psychotic Arousal as a Psychopathological Concept

By now, we presented several clinical observations, backed up by neurobiological findings pointing to the call for the psychopathological reconsideration of the role of emotions in the transition and establishment of psychosis. The clinical observation of a complex emotional state, characterized by an increased affective tone with concomitant symptoms of an experiential alienation, could be regarded as a psychopathological entity distinct from core psychotic symptoms such as delusions and hallucinations. The recognition and further investigation of this emotional state from psychopathology seems vital for translational research and correspondence with neurobiological findings.

A fundamental inquiry before we continue our study should regard the nature of abnormal experiences. For long, psychopathology studies have acknowledged the contribution of abnormal experiences to the formation of delusions, but hardly ever asked about their nature, implicitly regarding them as a kind of “perceptual disturbances” or unspecified “psychotic experiences”, and implicitly as “thoughts” being beyond the means of any other investigation unless philosophical [67,68,69,70,71,72,73,74,75,76]. However, these experiences cannot be regarded “perceptual” with the notion we apply to the term, since they do not concern any specific sensorial modality, nor can they be considered merely “thoughts” based on their quality characteristics and traits such as the varying degree of intensity and frequency with which they appear. Patients regard them as feelings, and when we examine them, we implicitly adopt this term to be intelligible to them. Even when we question delusional ideas using standardized questionnaires, we frequently adopt the term “Do you feel...” [77], but again, considering them as mere feelings could not easily correspond to our mainstream concepts about affective feelings.

Advancing the role of emotions in the psychotic continuum offered us the chance to reconceptualize the nature of abnormal experiences. In a previous published article [54] devoted extensively to the subject, we subsequently explored their nature and ventured to delineate their phenomenological characteristics. These abnormal subjective experiences, though not perceptual in their nature, color perceptual objects and events with undefinable and uncanny quality characteristics, in various intensities and a spontaneous mode. At the same time, these experiences enwrap the experiential events and objects with a highly self-referential significance and, depending on their severity, possess a quality of absolute truth and knowing. This “knowing” tends to defy the common knowledge/laws on which common reality experience and self-consciousness is based.

Based on the clinical observations and considering neurobiological evidence and theories, we conceptualized “abnormal experiences” mostly as of affective primarily origin, best described as “abnormal experiential feelings.” According to neuroscientific theories, these feelings are linked to primordial feelings that instantly and normally accompany any contact with surrounding objects, persons, the world, or related to the body itself [78,79,80,81]. We formalized their origin as “primary experiential feelings” derived by the re-representation of homeostatic/interoceptive feelings as they interact/commingle with external and internal images according to Damasio’s theory on the formation of basic consciousness [82,83,84,85].

Ordinarily, these feelings are always present and go unnoticed, but form the ground of basic consciousness from which the sense of subjectivity is derived [86]. Hence, abnormal experiential feelings may not be considered conjured up, but a deviant and an unpredictable outcome of an otherwise normal process: that of the emergence of normal experiential feelings that constantly and implicitly arise as the brain interacts with environmental or mental stimuli. As we also assumed, it is their affinity to the formation of basic consciousness that leads to a dysfunctional emergence of subjectivity and “self” as it appears in the acute psychotic episode reflected in specific thematic content. Patients describe their abnormal experiential feelings with words primarily expressing a rather limited repertoire of self-referential/transformative content of the self and the world, of losing the boundaries of the self or of the ownership of thoughts/movements or parts of the body and they further use this disturbed primary experiential content to form more sophisticated ideas implicating also the process of rationalization. These primary experiential feelings are the constitutional bricks that common rationale of reality experience is based on. No matter how intelligent, the person cannot dispute the truth of the specific experiential value they expose, although they often consider that others do not empathize with their view. However, not all events/objects/mental images are affected, since disturbed feelings are presented spontaneously at varying intensities and frequencies. Nevertheless, the process eventually triggers a cascade of neurocognitive events with deleterious effects on reasoning, memory, language and perception. Thus, we emphasize that the psychopathological manifestation of psychotic emergence explicitly provides a clinical paradigm of a disturbed formation of core consciousness and can serve for the evaluation of relevant neurobiological models. Even more, we stress that the neurobiology of schizophrenia should consider these psychopathological observations and refine the proposed models accordingly.

In the aforementioned publication [54], we described an emotional state, distinct as for the nature and long-term course from the ideational pictorial elaboration, though it shapes its core content and provides its affective load with appropriate certainty. We also hypothesized that it corresponds, at variable frequencies and intensities, to the emergence and acute phase of psychosis. Likewise, we have attributed it to a specific psychopathological construct, which we have termed “psychotic arousal.” “Psychotic arousal” can hence be defined as a diffuse affective state, dominated by these abnormal experiential feelings signifying disturbed self-processing while the brain interacts with external or internal objects, resulting eventually in experiential alienation of internal and external reality. Consequently, the concept of “psychotic arousal” is primarily a psychopathological construct that encompasses the affective turmoil felt by patients during the psychotic emergence and acute state, dominated by abnormal experiential feelings traditionally being part of the “abnormal psychotic experiences”. The clinical picture is further complemented by affective reactions responding to various cognitive and social consequences of the process.

### 1.3. The Study

#### Aims of the Study

This study is the first exploratory study of the major assumptions of the above conceptualization and namely the following:

- Psychotic arousal is a psychopathological construct distinct from other psychopathological phenomena and eligible for investigation;
- Abnormal experiential feelings are an integral part of this entity and are present during an acute schizophrenia episode;
- Psychotic arousal is amenable to antipsychotic treatment during the first period of treatment.

We also predict that in the acute phase of the disorder, it will exhibit a high affinity with other affective measures, and that its severity would have an impact on the certainty with which delusions are held.

To explore the above assumptions, we first developed an assessment scale for the investigation of psychotic arousal—The Psychotic Arousal Scale (PAS)—and we proceeded with an initial inspection of its psychometric properties. Consequently, we explored its relation to other psychopathological measures, and we tested its sensitivity to change in the course of antipsychotic treatment in an acute psychotic episode.

## 2. Materials and Methods

### 2.1. The Scale

#### 2.1.1. Development of the Scale

For the development of the scale, we took into consideration best practices on scale development, elaborated and summarized by Boateng et al. [87].

Several years of carefully monitoring patients while acutely ill and overcoming an acute psychotic episode provided an extended pool of abnormal experiential feelings, dominating the emotional state of the patients, most of them powerful enough to alienate perceived reality and sustain delusional beliefs. Our clinical work on a crisis intervention unit for severe mental disorders [88] provided the opportunity to monitor, through clinical interviews, dozens of patients with an acute psychotic episode, either first or recurrent, following their improvement while in treatment and repeatedly checking our observations. The abnormal experiential feelings included in the inventory correspond to cumulative reports of patients’ descriptions regarding their abnormal feelings. These feelings are eventually capable of supporting abnormal beliefs and misattributions when patients are clearly psychotic, but many of them—even if active psychosis is present—are not interpreted accordingly. Nevertheless, patients recognize the peculiarity of their feelings and their abrupt appearance, though rarely address them unasked as pathological. We approached these feelings as the minimal and primary condition, common to the vast majority of emergent psychotic manifestations that can be found on the ground of delusional beliefs and regardless of the secondary narrative and pictorial elaboration (e.g., elaborated ideas of reference entail sudden feelings of self-relevance in random incidents, and ideas of thought broadcasting may entail feelings of not being distinct or delimited as a unique organism or mind, as well as ideas of thought insertion may entail both the previous condition plus the loss of normal feelings of ownership of own thoughts).

To generate a representative set of items relevant to these “primary experiential feelings”, we also turned to other writers’ work, who meticulously and substantially delve into the area of subjective psychotic experiences. We especially mention Chapman’s inspirational work on the early symptoms of schizophrenia [89,90], as well Cutting’s work on subjective experiences in psychosis [91] and the symptom checklist for the examination of anomalous self-experience (EASE scale) by Parnas and his colleagues [92]. Although at least our formulation of psychotic arousal is diverging from their concepts in that it emphasizes the emotional primacy for these experiences in the context of psychotic emergence and highlights its course, including—at its peak—the active delusional phase and not only the pre-delusional period, it is their extended work that stimulated us and provided the rational basis for this exploration.

Furthermore, we attributed major importance to the way the items are expressed to be intelligible for the patients themselves and as close as possible to their experiences, minimizing the need to analyze further an item to the patient, however absurd the statements could be for someone that never experienced such feelings. Conceptualizing these “primary experiential feelings” as really present and potentially valid enough for patients to effectively result in a reality transformation, we approached them accordingly; therefore, we adopted an affirmative, straightforward expression instead of an explorative one.

Apart from the dimension of the primary abnormal experiential feelings, the inventory includes a dimension for secondary or ancillary feelings in response to cognitive and operational shortfalls accompanying the emergence of the disorder. It also includes a third dimension related to general anxiety feelings corresponding to common anxiety feelings and affective reactions to detailed delusional ideas and hallucinations, and other symptoms.

In order to evaluate whether generated items as well as the pre-specified dimensions adequately represent the construct of psychotic arousal as we approached it, we conducted the Delphi method [93] with 5 experts (academic and non-academic psychiatrists with demonstrated interest in psychopathology). Several rounds of the initial questionnaire were conducted following an extended initial communication, with appropriate informational material that ended with agreement after the final session. Before the establishment of the preliminary inventory, we cautiously cross-validated the coherence and comprehension of the initial items with several patients (N: 10), and we made appropriate modifications.

#### 2.1.2. Description of the Scale

The initial scale (see Appendix A) includes 3 pre-specified dimensions of subjectively experienced affective symptoms (64 items in total):

- “Primary experiential feelings” (non-exhaustive list of abnormal experiential feelings relative to instinctual conception of the self, others, or world images) (45 items);
- “Secondary/Ancillary feelings” (feelings associated with cognitive and operational shortfalls accompanying the emergence of the disorder) (10 items);
- “General anxiety feelings” and vague affective reactions relative to erroneous beliefs or perceptual disturbances and other consequences of the disorder (9 items).

#### 2.1.3. Administration of the Scale

It has highly been suggested before the administration of the questionnaire to precede this with contact with the patients, showing a more caring attitude than a neutral approach. This takes into consideration their own feelings and standpoint of their condition, highlighting the specific attention of the interview on the unusual feelings experienced by patients during the identified interval. Generated items were in general comprehended with ease and, surprisingly, patients responded to questions expressing a rather revealing feeling of being understood. Answers were easily elicited by patients who offered additional details and plenty of paradigms in the case a symptom was present, while denying without doubt its presence when this was the case. For both situations, clarifying examples were instructed to be required either from the subject or the examiner.

Interestingly, as soon as the patient understood the objective of the questions, answers became fluent and with high certainty. One thinks that if a patient is properly educated in considering these feelings, the inventory could be completed by the patient alone. However, very disorganized patients are not able to collaborate and should be avoided until disorganization is resolved. In any case, the requirements for the administration of the instrument are an adequate intellect, free willingness to participate and a sufficient capacity to organize their thoughts. However, disturbances in concentration being very prominent at the selected time of the interview and the consideration of the challenges involved implied the need—and it is also recommended—that mental health professionals familiarized with these psychotic manifestations administer the inventory.

The time reference of investigation was set on the previous ten days, and it examined the presence of the symptoms in the specific time interval in two dimensions: Frequency and Intensity.

Frequency: how often the symptom is present if at all, in a Likert scale from 0–5:

0 Doubtful or absent;
1 Rare: 1–2 times (on different days) within the previous 10 days;
2 Moderate: 3–4 times (on different days) within the previous 10 days;
3 Moderately Severe: almost daily, very few days free of symptoms (1–3 days) within the previous 10 days;
4 Severe: daily (Some days only 1 or 2 instances albeit every day);
5 Very Severe: daily (very frequently—multiple times within the same day, every day).

After the Scoring of the Frequency for each item, if present, we proceeded to the evaluation of the Intensity. The scoring of intensity is based on the degree of the intensity the patient has attributed to the feeling within the previous ten days. On the scale (1–10), 1 corresponds to the minimal (subtle) intensity and 10 to the maximum intensity. According to the instructions, the maximum degree of intensity during the respective time interval is noted.

A composite score (sum) taking into consideration both frequency and intensity is derived for each item. The approximate time to complete the questionnaire is 20–25 min depending on patients’ ability to concentrate.

### 2.2. The Pilot Study

#### 2.2.1. Sample of Patients

Consecutive patients (N: 66) experiencing an acute psychotic episode, either first or recurrent in the context of schizophrenia or schizophreniform disorder, comprised the initial sample of our research protocol. Exclusion criteria were any intellectual disability, organic pathology relative to psychiatric manifestations and substance use. Diagnosis was confirmed by two psychiatrists according to DSM-5 criteria, history and clinical evaluation during the period of therapeutic intervention and follow up (the clinical follow up of the patients extended for six more months for purposes related to another research protocol). Seven patients remained very disorganized during the first week of admittance and were excluded from the initial sample. Additionally, four patients out of the sixty-six finally fulfilled criteria for non-schizophrenic psychosis (three in the affective spectrum and one due to an organic disorder, undiagnosed at the entrance), and were therefore excluded from the final sample. The final sample ultimately consisted of 55 patients recruited from the Crisis Intervention Unit for severe mental disorders of the 1st dept. of Psychiatry, University of Athens, Medical School (N: 45 outpatients) and (N: 10 inpatients) voluntary recently hospitalized in a Psychiatric ward of “Sismanoglion General Hospital”.

Interviews were conducted as close to admittance to the unit or ward as possible and as soon as the patient could collaborate adequately (by instructions, as soon as practicable, no more than 7 days after admission). Since the study was a naturalistic study, there was some variation in treatment status. Some patients had begun taking medication several days before entrance into the unit or clinic, while others had not. In either case though, symptomatology was considered severe enough to imply either hospitalization or specific intervention. The study was approved by the Ethics Committee of both hospitals and the participants were patients who freely agreed to participate and signed an informed consent statement.

The interviewers were 3 psychiatrists (PA, IV and MS), fully conversant with all scales under study, who were checked for their inter-rated reliability on all the scales used (live interviews with interchanged interviewers and independent scoring, following strict instructions to avoid parallel clarifying questions). Inter-rater reliability coefficients, specifically for PAS, in a sample of 18 patients revealed excellent consistency (absolute agreement) between the three raters in a two-way mixed effects model (ICC’s: 1.00 with lower bound of confidence interval at 95%: 1.00), justified by the minimum requirement for interpretation by the raters of the patients’ answers, while the ICCs for the remaining scales ranged from 0.88 to 0.93.

#### 2.2.2. Scales

1. The Psychotic Arousal Scale (PAS) (initial form) (64 items);
2. The Positive and Negative Syndrome Scale (PANSS): a standardized, clinical interview that rates the presence and severity of positive and negative symptoms, as well as general psychopathology for people with schizophrenia [94,95];
3. The Brown Assessment of Beliefs Scale (BABS): a semi-structured, rater-administered scale that assesses insight/delusionality in a variety of disorders [96];
4. Hamilton Anxiety Scale (HAM-A): a clinician-rated scale developed to measure the severity of anxiety symptoms in both clinical and research settings [97];
5. The Calgary Depression Scale for Schizophrenia (CDSS): a nine-item clinician-rated measure that assesses the level of depression in people with schizophrenia [98,99,100];
6. Barnes Akathisia Rating Scale (BARS): a rating scale administered by clinicians to assess the severity of drug-induced akathisia [101,102];
7. WAIS-R: short form (the vocabulary, block design scores, and the estimated full-scale score were calculated) [103,104,105].

The study includes the following:

(a) A primary analysis on the internal reliability of the scale.
(b) An initial exploration on the relationships with global psychopathological measures, anxiety/depression measures and with the Barnes Akathisia Rating Scale. The latter scale was chosen as a discriminant index for anxiety and uneasiness due to antipsychotic treatment.
(c) Since our hypothesis was that psychotic arousal characterizes prominently the acute phase of psychosis and is subject to the action of psychopharmacological agents during the first period of therapy, we also tested sensitivity to change on a sample of crisis intervention patients. Taking into consideration the overall mean time of the intervention in the unit published elsewhere [88,106], we tested patients on PAS at three consecutive periods (T1: at entrance, T2: 15–20 days later, and T3: at discharge).

#### 2.2.3. Statistical Analysis

Quantitative variables were expressed as the mean (standard deviation) or median (Inter-Quartile Range), and qualitative variables were expressed as absolute and relative frequencies. Student’s *t*-tests were computed for the comparison of mean values. Internal consistency reliability was determined by the calculation of Cronbach’s α coefficient. Reliability scores equal to or greater than 0.70 were considered acceptable. Spearman correlation coefficients (rho) or Pearson correlation coefficients (r) were used to explore the association of two continuous variables. To examine the sensitivity of PAS scale to change, mixed linear regression models were applied with dependent variables being the PAS scores. Regression coefficients (β) with standard errors (SE) were computed from the results of the mixed models. All reported p-values are two-tailed. Statistical significance was set at <0.05 and analyses were conducted using SPSS statistical software (version 26.0).

## 3. Results

The final sample included 55 participants (58.2% men) with mean age 37.8 years (SD = 11.3 years). Fourteen (14) patients out of the total were displaying a first psychotic episode. Their characteristics are presented in Table 1.

The subscale of the “primary experiential feelings” initially consisted of 45 items. However, items 2, 5, 8, 10, 30, 33, 34, 38, 43, 44 and 50 were excluded from it due to the low corrected item–total correlation (less than 0.3). The corrected item–total correlation and Cronbach’s α coefficient (if item deleted) are presented in Table S1 (Supplementary Materials). Overall, Cronbach’s a coefficient was 0.91. Subsequently, the examination of the Inter-Item Correlation via Pearson’s correlation coefficient revealed that item 16 was highly correlated with items 15 (r = 0.84) and 20 (r = 0.84), and item 21 was highly correlated with item 17 (r = 0.78). Thus, items 15, 20, and 17 were also excluded from the primary experiential feeling’s subscale, and the reliability of the scale was checked again. The remaining items (N: 31) had acceptable corrected item–total correlation (greater than 0.3) and total Cronbach’s a coefficient was 0.90, which was also acceptable. The mean score for the dimension of “primary experiential feelings” was 92.71 (SD = 72.07).

The “secondary/ancillary feelings” subscale consisted of 10 items. All items had an acceptable corrected item–total correlation (greater than 0.3) and the total Cronbach’s a coefficient was 0.90, which was also acceptable (Table S2 in Supplementary Materials). The mean score for this dimension was 50.35 (SD = 37.75).

The subscale of the “general anxiety feelings” consisted of nine items. All items had an acceptable corrected item–total correlation (greater than 0.3) and the total Cronbach’s a coefficient was 0.78, which was also acceptable (Table S3 in Supplementary Materials). The mean score for the general anxiety feelings subscale was 54.68 (SD = 29.8).

The mean total PAS score was 197.74 (SD = 122.85). Cronbach’s α coefficient for the PAS total score was acceptable and equal to 0.94.

The descriptive statistics for all scales under study are presented in Table 2.

*t*-test analysis on the PANSS total score among inpatients (80.64 ± 25.33) and those from the crisis intervention unit for severe mental disorders (70.69 ± 15.64), indicated non-significant differences in illness severity t (53) = 5.050, *p* = 0.089. 

PAS total scores were not associated significantly with sex (*p* student’s *t*-test: 0.898), or between those with a duration of illness less than one year and the rest of the patients (*p* Student’s *t*-test: 0.945), as well as between those with a first psychotic episode and the rest of the patients (*p* student’s *t*-test: 0.900). PAS total scores were not significantly associated with patients’ age (r: −0.11, *p*: 0.421), educational years (r: −0.21, *p*: 0.161), number of hospitalizations (r: −0.16, *p*: 0.278), months of current disease (r: 0.15, *p*: 0.303) and years of disease (r: −0.08, *p*: 0.592). Moreover, PAS total scores were not significantly correlated with WAIS (r: 0.27, *p*: 0.215). 

All PAS subscales were significantly and positively correlated with each other, as well as with the PAS total score (Table S4 in Supplementary Materials).

Correlations of PAS and the rest of the scales under study are presented in Table 3. The PAS total score was modestly correlated with the PANSS total score, the PANSS Positive score, and the PANSS General Psychopathology score, as well as with the PANSS anxiety, depression and guilt items, along with the item of active social avoidance. Moreover, modest correlations were found between the PAS total score and its subscales with CDSS, while strong positive correlations were found between the PAS total score and subscales and the Hamilton anxiety scale. No correlations were found with the Barnes scale. Alongside that, the PANSS delusion item, the BABS total score and specifically its first item on conviction revealed significant positive correlations with the ‘primary experiential feelings’ subscale and/or the PAS total score.

To examine the sensitivity of the PAS scale to change, as we already mentioned in the Methods section above, we analyzed PAS scores in three consecutive time periods during acute phase intervention (Table 4). The first evaluation (T1) followed the patients’ entrance within 3 (2.76) days. The second evaluation (T2) followed 21 (6.48) days later, and the third evaluation, at discharge, (T3) 50 (26.69) days from the first evaluation. Twelve patients out of forty-five did not complete the final assessments in time, mostly for circumstantial reasons and unrelated to the treatment outcome. Most of them missed their scheduled appointment being outside the area of Athens at the time, or due to negligence on the part of their relatives. Therefore, we performed a one-sample z-test to calculate any differences of the means among the missing patients and the rest for PAS total scores and PANSS total scores at the first assessment time. The corresponding results were as follows: z statistic: −0.52, *p*-value: 0.59, Cohens-d: −0.17) and (z statistic: 0.00, *p*-value: 1, Cohens-d: 0.00.

**Figure 1:**
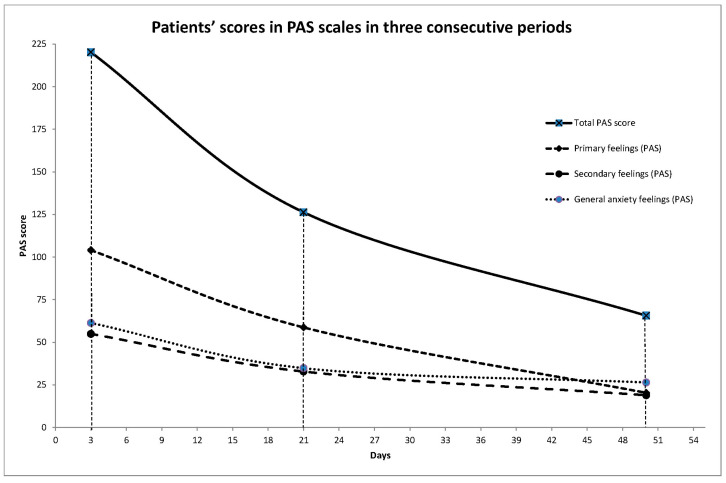
Patients’ scores of PAS scales for three consecutive periods.

Consequently, mixed linear regression models were applied to examine the difference in patients’ PAS scores at the three consecutive periods (Figure 1). The results are presented in Table 5. Significant reductions were found in all PAS subscales and the total score from T1 to T2 and from T1 to T3, while from T2 to T3, no significant changes were found.

## 4. Discussion

In this study, we present the initial exploration of a conceptually defined psychopathological construct aiming to portray psychotic arousal or otherwise the experiential emotional component of the acute psychotic state. The core aims of the study were to investigate the conceptual assumptions endorsed in the project that are subject to verification and further evaluation.

The main assumptions can be summarized as follows: (1) the experiential emotional state of a patient in acute psychosis, described herein as psychotic arousal, is a discrete state, eligible to investigation; (2) abnormal experiential feelings are an integral part of this state, distinct from other psychotic manifestations, such as delusions or hallucinations; and (3) the state is responsive to antipsychotic intervention during the first weeks of treatment. To pursue our target, we developed a scale accordingly, i.e., the Psychotic Arousal Scale (PAS), and we explored its initial psychometric properties.

Following our original hypothesis, the target population in this pilot study was patients with a schizophrenia disorder in an acute exacerbation of the illness or at a first schizophrenic episode.

Exploratory analysis on the internal reliability of the Psychotic Arousal Scale (PAS) proved satisfactory, with high internal consistency coefficients (0.90) for the dimensions of the “primary experiential feelings” and the “secondary feelings”, while for the third dimension of the “general anxiety feelings” there was a modest internal consistency coefficient (0.78). The overall internal reliability of the scale was excellent with a Cronbach α coefficient of 0.94.

All three dimensions of the scale highly correlated to each other and with the overall scale, suggesting that they are plausibly part of the same psychopathological construct.

Our anticipation that the PAS, being an estimation of the affective state of the patient during acute psychosis, should highly correlate with other affective measures, such as the depression and anxiety items of the PANSS, the Calgary Depression Scale, and the Hamilton Anxiety Scale, was also confirmed for all three of the PAS dimensions. Van Os [107] argued that depressive states and psychotic experiences may present a continuum, and many other scholars recently suggested that a common etiological mechanism may underlie the presentation of comorbid depression, anxiety and psychotic-like experiences in schizophrenia [108,109,110]. Our work is highly supportive of a link between affective symptoms (depressive and/or anxiety) and abnormal experiences in acute schizophrenia, as indicated in the current study, especially by their strong correlation with the “primary experiential feelings”. In fact, we argue that our suggestion that these subjective psychotic experiences should be regarded and studied as mere “abnormal experiential feelings” of an affective origin, is supported by the results of this first investigation.

PAS scores were not related to sex, age, years of education, duration of illness, number of hospitalizations or months of current disease. As it was anticipated, the Psychotic Arousal Scale significantly correlated with the PANSS total score, PANSS Positive score and PANSS General Psychopathology score, though not with the PANSS negative score, indicating, overall, its affinity mostly with the state-like psychopathological severity of acute psychosis.

Correspondingly, a strong positive correlation was found between the PAS total score and all three dimensions with the PANSS item on active avoidance. Indeed, anxiety has been repeatedly connected to avoidance behavior [111,112,113,114] and studies investigating lifetime occurrence of the anomalous self-experiences in early phases of psychosis suggest a negative contribution on overall social functioning, including social avoidance [115]. However, we should also consider the possibility of a more stringent relation among an alleged disturbance of a more primary affective network resulting in disordered decision making and avoidance [116].

Similarly, the PANSS item on delusions and the BABS scale (a measure of delusionality) correlated significantly with the dimension of “primary experiential feelings”, while both the total PAS score and “primary experiential feelings” significantly correlated positively with the item on conviction to the delusions of the BABS scale.

This study was not meant to explore in depth the contribution of psychotic arousal to delusional formation, but nonetheless, the results are indicative of a positive relation to delusions. Disturbed “primary experiential feelings” in the context of psychotic arousal are not equivalent to delusions; several abnormal experiential feelings are apparently present during the acute psychotic state without corresponding to specific delusions. Interestingly though, the degree of arousal—the intensity and severity of the “primary experiential feelings”, as well psychotic arousal overall—is positively related to the conviction with which patients hold delusional ideas.

Current and earlier explanatory models in the domain of cognitive neuropsychiatry stress the crucial role of abnormal experiences for delusion formation [57,117,118,119,120,121,122,123].

Early theories interpreted delusion as arising from an individual’s attempt to explain an abnormal perceptual experience, usually supposedly a neuropsychological anomaly able to justify the content of a specific delusion. The so-called “One factor account” for delusion formation, though providing an adequate explanation for the content of a delusion, could not explain numerous cases with the same putative deficits that did not develop a delusion, leaving also the question on the maintenance of the delusion unanswered.

To better explain these discrepancies in theory, researchers developed the model of the “two-factor account.” In this model, a second factor involving a non-specific deficit in belief evaluation, in conjunction with the first neuropsychological deficit, supposedly provides the necessary explanation for the maintenance of the delusion [124,125,126,127] (for a well-reasoned critique of these theoretical approaches on belief formation, see Connors and Halligan, 2020 [128]).

In our phenomenological inquiry, by focusing on the emotional arousal and its disturbed experiential feelings, we indicated an alternative perspective for the delusion formation without the need to rely on a second factor. Crucial importance is attributed to “primary experiential feelings”, repeatedly ascertaining their presence in clinical investigation, though they remain beyond any systematic exploration and distinct categorization as mere experiential phenomena in mainstream psychopathology. Mathew Rattcliff [129,130], who philosophically explored these feelings, favored the term “existential feelings” for them, stating that they “make a considerable contribution to the structure of experience, thought and action”. The results of this present study provide initial support that beyond shaping the content of delusional ideas—as already suggested—the degree of severity of psychotic arousal and abnormal experiential feelings is linked to the maintenance or the conviction with which delusions are held.

The next inquiry this study addressed was the clinical remark, repeatedly scrutinized in clinical practice, of the amelioration of this state in the course of antipsychotic treatment for the management of an acute psychotic episode before the overall symptomatic improvement, as already mentioned in the Introduction section. The examination of the sensitivity to change for the PAS scale during the treatment of the acute psychotic episode in three time periods (at the beginning, in the estimated middle and at discharge), revealed that though an altogether improvement was evident at the end of the treatment course, the maximum improvement, statistically significant, was observed during the first 2-to-3 weeks of treatment. Considering the naturalistic design of the study, the remaining time until discharge was apparently requisite for the amelioration of other disease characteristics—not addressed by this study—relevant to the disease process, and their overall compensation.

There are several limitations in this study. Before we proceed to the limitations, we must acknowledge that this research project is of an explorative type for a newly introduced psychopathological construct based on a hypothesis formulation, grounded on systematic clinical observation and respective pursuit in the literature.

The relatively small sample of patients included in the study limits the generalizability of results and the prospect for a more rigorous psychometric evaluation addressing the factorial analysis of the scale. Future employment, also of more rigorous psychometric testing, as the item response theory (IRT) over the classical test theory modeling used in this study could improve the reliability of the scale. Hence, the psychometric results of this study should only be considered as the first step in the psychometric analysis of the scale, though they are indicative and suggestive. Additionally, we did not opt for test-retest reliability analysis in this study due to the selection of patients in an acute state of illness and the anticipation of responsiveness of the evaluated symptoms to antipsychotic treatment. However, the absence of significant changes among T2 and T3 assessment suggests that the scale might present good test-retest reliability when patients are stable.

Furthermore, the naturalistic design of the study (evaluations that followed the natural course of typical clinical management), as well as the primary aims set by the study, did not permit for causal inferences among the parameters tested or an evaluation of the antipsychotic response dosage, but do encourage further research. In addition, following our initial hypothetical evaluation of the concept, we excluded other diagnoses with psychotic symptomatology apart from schizophrenia disorder from this study, as well as patients in different disease stages. Therefore, the results of this study could only be considered preliminary in the validation process of the hypothesis, testing the overall psychotic arousal concept. Future research should address these limitations by including larger samples of patients and testing psychotic arousal in different diagnostic categories and stages of the psychotic process. An interesting challenge would also be the parallel investigation of neurobiological and neurophysiological affective markers (cortisol, HRV, electrodermal skin conductance, etc.).

Nevertheless, there are several important inferences that can be derived from this study.

## 5. Conclusions

Addressing both the concept and the development of an instrument to assess psychotic arousal, we provided a psychopathological construct as close to the patient’s experience of acute psychosis and as close to the perceptive clinical evaluation of this experience as possible.

Initial assessment of the psychometric properties of the Psychotic Arousal Scale proved satisfactory. The scale seems to present good reliability and encourages its further standardization processing.

The results of this study, though preliminary, support the assumptions in test and encourage further research along this line. Namely, (a) abnormal experiential feelings (derealization/depersonalization feelings, feelings of self-relevance to trivial images or of losing the boundaries of self and ownership of thoughts/movements) are present during the acute psychotic state contrary to general unverified consensus that delimits their appearance in the pre-delusional state; (b) they share emotional traits such as intensity indicating the legitimacy of appraising them as of an emotional origin not considering the conceptualization offered in the introduction section; (c) early response of psychotic arousal to antipsychotic medication conforms to neurobiological findings and further underlines its distinct nature and its “state” character; (d) the degree of severity of the psychotic arousal and of abnormal psychotic experiences also seem to relate to the conviction of delusions providing an interesting exploratory path for the pathophysiology of the disease regarding the formation and maintenance of delusions.

Conclusively, the results of this exploratory study are suggestive of the presence of a continuum of an emotional arousal permeated by abnormal experiential feelings, in the emergence of acute psychosis largely neglected by contemporary psychopathology and provide the means for its evaluation. We stress the importance this reconceptualization could have for translational studies, mutually promoting the understanding of the biological underpinnings of psychosis and studies of consciousness in neuroscience. We also suggest that the concept of psychotic arousal provides the means to vigorously test the early efficiency of antipsychotic treatment and facilitate relative research evaluation.

Finally, it supports mutual understanding and communication with patients by recognizing the crucial role of disordered subjective experience in the pathology of the psychotic process. We strongly postulate that our future understanding on the physiological mechanisms that enable natural “feelings of what happens” and their subsequent pathophysiology will eventually facilitate communication with patients and advance therapeutic strategies.

## Figures and Tables

**Table 1:** Sample characteristics (N:55).

**Gender N (%)**		Men 32 (58.2)
		Women 23 (41.8)
Age, mean (SD)		37.8 (11.3)
Years of education, mean (SD)		14.5 (2.9)
Number of hospitalizations, mean (SD)		1.2 (2.2)
Months of current disease, mean (SD)		5.7 (10.8)
Years of disease, mean (SD)		10 (9.6)
Years of disease	≤1	13 (25.5)
	≤3	20 (39.2)
	≥4	31 (60.8)
Antipsychotic doses equivalent to 100 mg/day of chlorpromazine at discharge, mean (SD)		533.1 (198.7)
WAIS (full IQ estimated score)		100.1 (13.4)
Vocabulary score		11.7 (1.9)
Block design		9.7 (2.9)

**Table 2:** Patients’ scores for all scales under study.

Scale	Mean (SD)
**PAS**	
PAS total score	197.74 (122.85)
Primary feelings	92.71 (72.07)
Secondary feelings	50.35 (37.75)
General anxiety feelings	54.68 (29.8)
**PANSS**	
PANSS total score	73.15 (19.29)
PANSS positive score	19.62 (6.84)
PANSS negative score	14.79 (6.77)
PANSS general psychopathology score	38.27 (9.55)
PANSS/delusions	4.93 (1.36)
PANSS/hallucinations	2.96 (2.20)
PANSS/anxiety	4.25 (1.31)
PANSS/guilt feelings	2.56 (1.61)
PANSS/tension	3.33 (1.48)
PANSS/depression	2.64 (1.56)
PANSS/active social avoidance	2.93 (1.85)
**CDSS**	
CDSS total score	5.66 (6.07)
**HAMILTON-Anxiety**	
HAM-A total score	17.36 (11.63)
**BABS**	
BABS total score	14.25 (5.03)
babs1_conviction	2.80 (1.16)
babs2_perception of other views	1.63 (1.03)
babs3_explanation of differing views	2.44 (1.11)
babs4_fixity	2.40 (1.08)
babs5_disproval of ideas	2.75 (1.21)
babs6_insight	2.25 (1.24)
**BARNES Scale**	
Barnes total score	0.21 (0.51)

**Table 3:** Correlations of the PAS scale and subscales with all the under-study scales.

		Primary Feelings	Secondary Feelings	General Anxiety Feelings	Total PAS Score
PANSS Total score	r	0.37	0.46	0.35	0.44
	*p*	**0.02**	**0.00**	**0.03**	**0.01**
PANSS Positive score	r	0.35	0.11	0.41	0.34
	*p*	**0.02**	0.48	**0.01**	**0.03**
PANSS Negative score	r	0.03	0.25	−0.03	0.09
	*p*	0.83	0.10	0.87	0.50
PANSS General Psychopathology Score	r	0.39	0.54	0.43	0.50
	*p*	**0.01**	**0.00**	**0.01**	**0.00**
PANSS Delusion Item	rho	0.33	0.00	0.41	0.26
	*p*	**0.02**	0.98	**0.00**	0.08
PANSS Hallucination Item	rho	0.18	0.16	0.31	0.23
	*p*	0.23	0.30	**0.04**	0.12
PANSS Anxiety	rho	0.33	0.16	0.48	0.35
	*p*	**0.03**	0.30	**0.00**	**0.02**
PANSS Guilt	rho	0.38	0.46	0.33	0.47
	*p*	**0.01**	**0.00**	**0.03**	**0.00**
PANSS Depression	rho	0.39	0.44	0.38	0.46
	*p*	**0.01**	**0.00**	**0.01**	**0.00**
PANSS Active Social Avoidance	rho	0.34	0.55	0.29	0.49
	*p*	**0.02**	**<0.00**	**0.05**	**0.00**
CDSS Scale	rho	0.54	0.56	0.59	0.65
	*p*	**0.00**	**0.00**	**0.00**	**0.00**
HAM-Anxiety Scale	rho	0.66	0.53	0.68	0.72
	*p*	**0.00**	**0.00**	**0.00**	**0.00**
BABS Total Score	rho	0.34	0.04	0.21	0.27
	*p*	**0.05**	0.81	0.21	0.12
babs1 Conviction	rho	0.36	0.21	0.29	0.36
	*p*	**0.02**	0.19	0.07	**0.02**
Barnes Total Score (Akathisia)	rho	−0.08	−0.12	0.01	−0.14
	*p*	0.59	0.44	0.97	0.39

Notes. rho: Spearman’s correlation coefficient; r: Pearson’s correlation coefficient.

**Table 4:** Patients’ scores in PAS scales in the three consecutive periods.

	Primary Feelings (PAS)		Secondary Feelings (PAS)		General Anxiety Feelings (PAS)		Total PAS Score	
	N	Mean (SD)	N	Mean (SD)	N	Mean (SD)	N	Mean (SD)
T1	45	104.0 (73.4)	45	54.9 (39.2)	45	61.3 (27.9)	45	220.2 (122.8)
T2	18	58.7 (60.4)	18	32.8 (26.6)	18	34.8 (23.3)	18	126.3 (101.6)
T3	33	20.3 (30.2)	33	18.9 (24.1)	33	26.4 (24.6)	33	65.6 (71.2)

**Table 5:** Linear mixed-model results.

	T2 vs. T1		T3 vs. T1		T3 vs. T2	
	β (SE) ^1^	*p*	β (SE) ^1^	*p*	β (SE) ^1^	*p*
Primary feelings	−63.1 (11.7)	<0.001	−81.7 (9.2)	<0.001	−18.7 (12.4)	0.13
Secondary feelings	−27.8 (5.9)	<0.001	−36.0 (4.6)	<0.001	−8.3 (6.3)	0.19
General anxiety feelings	−30.6 (5.4)	<0.001	−34.6 (4.2)	<0.001	−4.1 (5.7)	0.48
Total PAS score	−124.9 (18.1)	<0.001	−152.2 (14.1)	<0.001	−27.3 (19.0)	0.15

^1^ regression coefficient (standard error).

## Data Availability

The datasets presented in this article are not readily available because the data are part of an ongoing study. Requests to access the datasets should be directed to the corresponding author.

## Supplementary Materials

The following supporting information can be downloaded at: https://www.mdpi.com/article/10.3390/jcm13185477/s1, Table S1. Item-total correlation coefficients and the Cronbach’s Alpha coefficients if item deleted for ‘Primary experiential feelings’ subscale. Table S2. Item-total correlation coefficients and the Cronbach’s Alpha coefficients if item deleted for ‘Secondary feelings’ subscale. Table S3. Item-total correlation coefficients and the Cronbach’s Alpha coefficients if item deleted for ‘General Anxiety feelings’ subscale. Table S4. Intercorrelations among PAS scales.

## Appendix A

Psychotic Arousal Scale (Initial form, 64 items with reference on excluded items and pre-specified dimensions)

1. Feels that the world around him/her is unwelcoming, dangerous and/or threatening (Primary)
2. Feels that other people are not the ones that they are supposed to be (as if they impersonate somebody else) (Primary) (Excl.)
3. Feels anxiety and fear of being threatened, intensified in places where there are other people (Primary)
4. Feels that unknown people on the street or television or dispersed talks refer to him/her (Primary)
5. Feels fear or insecurity that his/her physical integrity is at risk due to malicious acts of others (Primary) (Excl.)
6. Feels that his/her reputation is endangered by malicious acts of others (Primary)
7. Feels that other people pull a prank on him/her, make fun of him/her, mock him/her or play a trick on him/her (Primary)
8. Feels being monitored (Primary) (Excl.)
9. Feels being alone in the world (Primary)
10. Feels that others may unfairly accuse him/her for his/her acts or omissions (Primary) (Excl.)
11. Does not feel others as real or genuine (as if they are in disguise, fake or play a role) (Primary)
12. Feels somatic symptoms that worry him/her (tachycardia, somatic aches, breathing difficulties, skin itching etc.) (Gen. Anxiety)
13. Feels that his/her body or certain organs do not function properly or have undergone some distortion of their physical state (Primary)
14. Feels that someone can affect his/her body by distorting it in some way (Primary)
15. Feels that his/her somatic senses are the byproduct of someone or something else’s actions (they are caused by someone or something outside himself/herself) (Primary) (Excl.)
16. Feels that some parts of his/her body or organs or even his/her entire body are not his/hers (Primary)
17. Does not feel the existence of parts of his/her body or organs or even his/her entire body (Primary) (Excl.)
18. Feels that in order to make even simple movements for example walk or grasp something, he/she has to exert a lot of effort (Secondary)
19. Feels that the movements he/she makes are not his/hers (feels that he/she lacks the ownership of movements he/she makes (Primary)
20. Feels that the senses of his/her body are not his/hers (Primary) (Excl.)
21. Feels that his/her body or some parts of it (face, nose, limbs) have changed or are changing (Primary)
22. Feels his/her mind is confused (Secondary)
23. Feels difficulty in concentrating (Secondary)
24. Feels that he/she does not feel his/her feelings (Primary)
25. Feels difficulty remembering (Secondary)
26. Feels that he/she cannot easily make simple decisions (Secondary)
27. Feels that he/she has to exert effort in order to understand things once considered self-evident (Secondary)
28. Feels that his/her mind is empty or being emptied from thoughts (Secondary)
29. Feels that his/her mind is flooded by thoughts without being able to control it (Secondary)
30. Feels that words that once had no significance to him/her have now acquired a deeper meaning that is now important for him/her to understand (Primary) (Excl.)
31. Feels that his/her attention becomes easily distracted by unrelated things and he/she cannot control it (Secondary)
32. Feels that he/she must exert a lot of effort to do things he/she once did without thinking about it (Secondary)
33. Feels that he/she can hear his/her thoughts (Primary) (Excl.)
34. Feels that his/her mind abruptly stops thinking (Primary) (Excl.)
35. Feels that inside him/her, he/she is inanimate (Primary)
36. Feels that he/she is not in control of his/her mind (Primary)
37. Feels that some of his/her thoughts are not his/hers, but were in a way inserted in his/her mind (Primary)
38. Feels that the others can in some way “read” his/her thoughts (Primary) (Excl.)
39. Feels that his/her movements are being controlled from the outside (like a puppet on a string) (Primary)
40. Feels that his/her feelings are being controlled by someone else or something else (Primary)
41. Feels that he/she no longer feels himself/herself (Primary)
42. Feels that he/she has sinned and is guilty of something very important (Primary)
43. Feels that he/she has acquired or possesses special/supernatural abilities or forces (Primary) (Excl.)
44. Feels that his/her life has suddenly acquired a special significance/destination/mission (Primary) (Excl.)
45. Feels that the entire world around him/her has somehow or in some way changed (Primary)
46. Feels that the world around him/her and its constituents are not what they are supposed to be (as if they were a theatrical scenery) (Primary)
47. Feels that the world or objects that surround him/her are not real or genuine (as if they are fake) (Primary)
48. Feels that things around him/her once indifferent to him/her are not coincidental, and have a specific meaning that he/she cannot ignore and must definitely understand (Primary)
49. Feels that the world is mysterious and has a meaning he/she must discover (Primary)
50. Feels that living beings around him/her are inanimate (Primary) (Excl.)
51. Feels anxiety, fear or premonition that a big disaster has already or is about to happen (Primary)
52. Feels that there is a direct connection between him/her and the world around him/her; irrelevant events acquire special personal meaning, become “signs” to him/her (Primary)
53. Feels that whatever he/she thinks may happen to the world (Primary)
54. Feels that there is no separating boundary between himself/herself and the others; feels unprotected, exposed, almost transparent against the intentions and moods of others (Primary)
55. Feels that there is no boundary between him/her and the world; whatever lies inside him/her is reflected on the outside and vice versa (Primary)
56. Feels anxiety and fear attributed to existing hallucinatory experiences and their content (auditory, visual, olfactory, tactile etc.) (Gen. Anxiety)
57. Feels anxiety, fear, anger, rage or sorrow related to the content of the delusional ideas and beliefs (for example, is afraid to be hospitalized holding that doctors are secret agents or is afraid to eat his/her mother’s food holding that she intends to poison him/her or feels rage over his/her persecutors) (Gen. Anxiety)
58. Feels anxiety and uneasiness that he/she cannot attribute somewhere (Gen. Anxiety)
59. Feels anxiety and fear for real problems (health, financial, etc.) (Gen. Anxiety)
60. Feels uneasiness for possible future problems (uneasiness in the possibility of a beloved person’s illness, his/her own (possibility of) illness or a possible financial catastrophe, etc.) (Gen. Anxiety)
61. Feels a feeling of inner tension and strain (Gen. Anxiety)
62. Feels anxiety and uneasiness vaguely attributed to “what is happening to him/her”, including difficulties in his/her thinking and general functioning (Gen. Anxiety)
63. Feels anxiety, sadness or disappointment related to the fact that the others do not empathize with what is happening to him/her (Gen. Anxiety)
64. Feels feelings that he/she describes as surprise, ecstasy, astonishment or bewilderment and puzzlement directly related to his/her experiences (Primary)
