# Supplementary material for: Psychotic Arousal and the Psychopathology of Acute Schizophrenia: An Exploratory Study of the Experiential Emotional State in Acute Psychosis
Source: J Clin Med. 2024 Sep 15;13(18):5477. doi: 10.3390/jcm13185477 (PMC11432037; doi:10.3390/jcm13185477)
Supplement: Supplementary file 1 [file jcm-13-05477-s001.zip › jcm-3170897-supplementary.pdf]

Supplementary File

Table S1. Item-total correlation coefficients and the Cronbach's Alpha coefficients if item deleted for 'Primary experiential feelings' subscale.

After removing items with Corrected Item-Total Correlation <0.3			After removing items with Corrected Item-Total Correlation<0.3 and items being highly correlated with each other	
**PAS item**	**Corrected Item-Total Correlation**	**Cronbach's Alpha if Item Deleted**	Corrected Item-Total Correlation	Cronbach's Alpha if Item Deleted
1	0.49	0.90	0.51	0.89
3	0.44	0.90	0.45	0.89
4	0.59	0.90	0.60	0.89
6	0.32	0.91	0.33	0.90
7	0.36	0.91	0.39	0.89
9	0.38	0.91	0.40	0.89
11	0.33	0.91	0.33	0.89
13	0.53	0.90	0.52	0.89
14	0.42	0.91	0.41	0.89
15	0.42	0.91	-	-
16	0.57	0.90	0.50	0.89
17	0.61	0.90	-	-
19	0.56	0.90	0.53	0.89
20	0.57	0.90	-	-
21	0.56	0.90	0.50	0.89
24	0.39	0.91	0.41	0.89
35	0.63	0.90	0.61	0.89
36	0.50	0.90	0.51	0.89
37	0.41	0.91	0.37	0.89
39	0.47	0.90	0.43	0.89
40	0.49	0.90	0.47	0.89
41	0.59	0.90	0.57	0.89
42	0.37	0.91	0.39	0.89
45	0.63	0.90	0.60	0.89
46	0.31	0.91	0.32	0.90
47	0.39	0.91	0.35	0.89
48	0.50	0.90	0.47	0.89
49	0.41	0.91	0.40	0.89
51	0.41	0.91	0.40	0.89
52	0.36	0.91	0.39	0.89
53	0.37	0.91	0.42	0.89
54	0.39	0.91	0.47	0.89
55	0.31	0.91	0.37	0.89
64	0.42	0.91	0.40	0.89

Table S2. Item-total correlation coefficients and the Cronbach's Alpha coefficients if item deleted for 'Secondary feelings' subscale.

PAS item	Corrected Item-Total Correlation	Cronbach's Alpha if Item Deleted
18	0.45	0.90
22	0.70	0.88
23	0.78	0.88
25	0.58	0.89
26	0.71	0.88
27	0.63	0.89

28	0.46	0.90
29	0.67	0.88
31	0.70	0.88
32	0.71	0.88

Table S3. Item-total correlation coefficients and the Cronbach's Alpha coefficients if item deleted for 'General Anxiety feelings' subscale.

PAS item	Corrected Item-Total Correlation	Cronbach's Alpha if Item Deleted
12	0.31	0.78
56	0.49	0.75
57	0.51	0.75
58	0.55	0.75
59	0.53	0.75
60	0.36	0.77
61	0.54	0.75
62	0.55	0.75
63	0.35	0.77

Table S4. Intercorrelations among PAS scales.

	Secondary feelings	General anxiety feelings	Total PAS score
Primary feelings	0.64	0.66	0.94
Secondary feelings	1.00	0.56	0.82
General anxiety feelings		1.00	0.80

Note. All coefficients were significant at p<.001
